# A new lipid-rich microalga *Scenedesmus* sp. strain R-16 isolated using Nile red staining: effects of carbon and nitrogen sources and initial pH on the biomass and lipid production

**DOI:** 10.1186/1754-6834-6-143

**Published:** 2013-10-06

**Authors:** Hong-Yu Ren, Bing-Feng Liu, Chao Ma, Lei Zhao, Nan-Qi Ren

**Affiliations:** 1 State Key Laboratory of Urban Water Resource and Environment, Harbin Institute of Technology, Harbin 150090, China

**Keywords:** Biodiesel, Heterotrophy, Isolation, Lipid, Microalga, Nile red

## Abstract

**Background:**

Biodiesel production from oleaginous microalgae shows great potential as a promising alternative to conventional fossil fuels. Currently, most research focus on algal biomass production with autotrophic cultivation, but this cultivation strategy induces low biomass concentration and it is difficult to be used in large-scale algal biomass production. By contrast, heterotrophic algae allows higher growth rate and can accumulate higher lipid. However, the fast-growing and lipid-rich microalgae that can be cultivated in heterotrophic system for the industrial application of biodiesel production are still few. Traditional solvent extraction and gravimetric determination to detect the microalgal total lipid content is time-consuming and laborious, which has become a major limiting factor for selecting large number of algae specimens. Thus, it is critical to develop a rapid and efficient procedure for the screening of lipid-rich microalgae.

**Results:**

A novel green microalga *Scenedesmus* sp. strain R-16 with high total lipid content was selected using the Nile red staining from eighty-eight isolates. Various carbon sources (fructose, glucose and acetate) and nitrogen sources (nitrate, urea, peptone and yeast extract) can be utilized for microalgal growth and lipid production, and the optimal carbon and nitrogen sources were glucose (10 g L^-1^) and nitrate (0.6 g L^-1^), respectively. Compared to autotrophic situation, the strain R-16 can grow well heterotrophically without light and the accumulated total lipid content and biomass reached 43.4% and 3.46 g L^-1^, respectively. In addition, nitrogen deficiency led to an accumulation of lipid and the total lipid content was as high as 52.6%, and it was worth noting that strain R-16 exhibited strong tolerance to high glucose (up to 100 g L^-1^) and a wide range of pH (4.0-11.0).

**Conclusions:**

The newly developed ultrasonic-assisted Nile red method proved to be an efficient isolation procedure and was successfully used in the selection of oleaginous microalgae. The isolated novel green microalgal strain R-16 was rich in lipid and can live in varied and contrasting conditions. The algae appeared to have great potential for application in microalgae-based biodiesel production.

## Background

Conventional fossil fuels, such as petroleum, coal and natural gas, still play a dominant role in the global energy consumption [1]. However, it is well known that these traditional fuels are non-renewable with depleting reserves and increasing cost [2,3]. The most promising method to meet the growing demand for energy is to explore economically feasible and alternative fuels [4,5]. Recently, biodiesel exhibits great potential and attracts extensive interest as it is carbon-neutral and environment friendly [6]. Traditional feedstock of biodiesel contains plant oils (canola, corn, soybean, oil palm, coconut, etc.) and animal fats [7]. Nevertheless, such raw materials may compete with food supply, increase the utilization of limited farmland, and require long time to harvest which is hard to satisfy the large and long-term global energy demand [8].

Nowadays, biodiesel production technology from microalgae is widely considered as a potential and efficient method since a number of advantages, such as the simple cellular structure of microalgae, short production cycle, high intracellular lipid content, and fast growth rate [8,9]. In addition, microalgae can be cultivated on non-arable land which could reduce the demand for farm-land and avoid the competition with food/feed crops [10]. Though many microalgal strains have been isolated and established to be rich in neutral lipid, there are still some unknown species or strains present in various local environments with the potential of applying in the production of biodiesel.

Microalgal biomass production has largely been obtained by autotrophic cultivation in open pond or closed photo-bioreactor under natural or artificial source of light [11,12]. Nevertheless, the cell density of this culture strategy is low and the light requirement is high, and these bottlenecks make it hard to be applied in large-scale algal biomass production [11]. Compared to photoautotrophy, heterotrophic cultivation allows higher algal growth rate and enables microalgae to accumulate higher biomass and amounts of lipid using less time in the absence of light, which is critical for reducing the microalgal biomass production cost [13]. However, only a few microalgae species adapt to heterotrophic cultivation, and most of them belong to the genus *Chlorella*[12]. As such, it is important to screen more fast-growing and lipid-rich microalgae that can be cultivated in heterotrophic system for the industrial application of biodiesel production.

On the other hand, current methods to determine the total lipid content for selecting large number of algae specimens are complicated and generally contain extraction, purification, concentration and quantification of lipids [14]. This process is laborious and the lipid components decompose easily [15]. Recently, rapid and efficient screening methods are essential to decide novel and proper algae candidates. Nile red is a lipophilic fluorescent dye and has been used in algal biodiesel production to detect and quantify the lipid content of many microalgal strains [12]. Furthermore, some researchers further modify the Nile red fluorescence technique to improve the lipid staining efficiency and obtain satisfactory outcomes [15,16]. However, up to now, the information about using the modified Nile red method for screening of lipid-rich microalgal species or strains is still limited.

So, this study developed a novel ultrasonic-assisted staining procedure for algal sample analysis. The isolated microalgal strain was further investigated in heterotrophic cultures for lipid production with high efficiency. Moreover, the main factors (carbon source, nitrogen source and initial pH) were systematically studied and optimized for high biomass and lipid production. In addition, this work also compared the lipid and biomass production ability of the selected strain with those previously reported.

## Results and discussion

### Algal isolation with improved Nile red method

**Figure 1:**
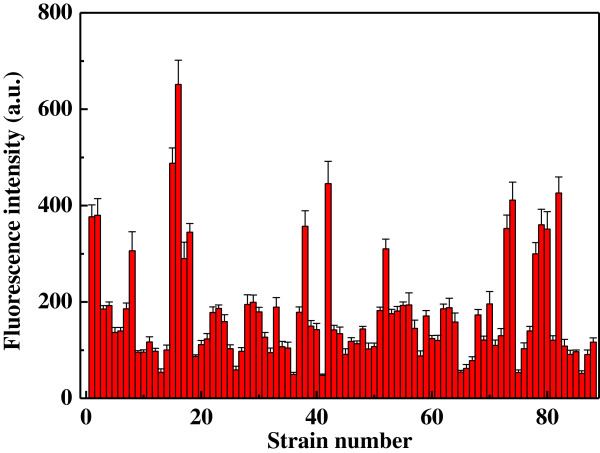
Fluorescence emissions of 88 algal strains obtained with the ultrasonic-assisted Nile red method.

BG-11 medium supplemented with glucose was frequently used for the cultivation of green algae [17]. Eighty-eight strains of microalgae like microorganisms that can grow on BG-11 agar plate were selected using the above mentioned produces. It was found that all the 88 colonies can grow well on the BG-11 agar plate and exhibited green color, which implied that all the isolated strains belonged to the division of Chlorophyta. In addition to cell growth, total lipid content was another critical indicator to decide whether the screened algal strain was a promising lipid producer with high lipid production ability. Solvent extraction and gravimetric determination was the most commonly used method to ascertain the total lipid content of microalgae [16]. However, this traditional method is time-consuming and requires large amounts of microalgae samples, making it difficult to be applied in the rapid and efficient screening of lipid-rich algal strains [14]. Nile red is a lipid-soluble dye and has been widely employed to determine the cellular lipid content of microalgae in qualitative and in situ quantitative analysis [15]. Nevertheless, most green algae possessed rigid cell wall boundaries which may inhibit the Nile red from dissolving in the cellular lipid [16]. Therefore, a useful and effective ultrasonic-assisted method was applied to stain the lipid compounds in the present work. The fluorescence emissions of 88 algae isolates were obtained using the Nile red method (Figure 1). Seventy-five algae isolates gave weak fluorescence intensity (less than 300 a. u.), and high fluorescence intensities achieved from the other 13 isolates indicated the high lipid content. In particular, the strain R-16 yielded quite strong fluorescence intensity (651 a. u.), suggesting it was a promising alternative feedstock for the production of lipid.

### Identification of the microalga

**Figure 2:**
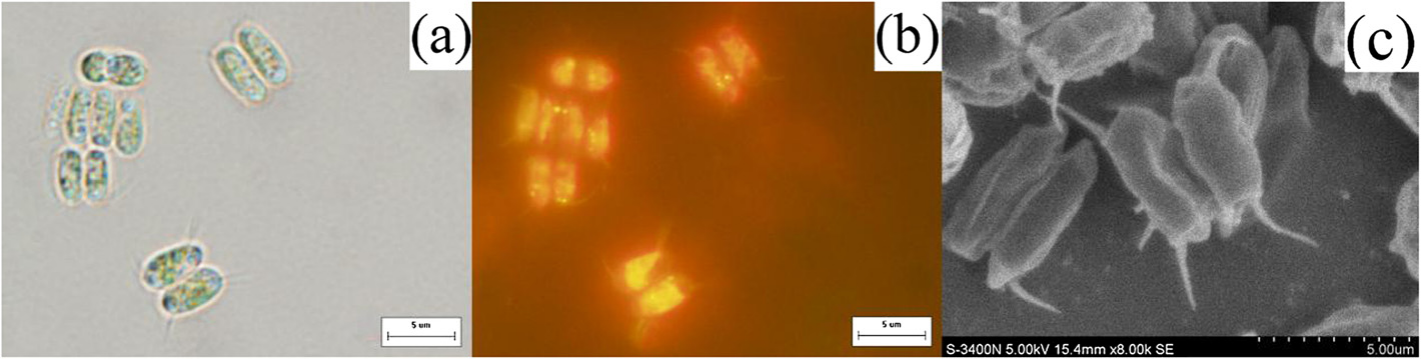
**Microscopic morphological observation and SEM image of strain R-16. (a)**, light micrograph; **(b)**, fluorescence micrograph of microalgae stained with Nile red; **(c)**, SEM image (8,000 ×).

The microalgal strain R-16 was selected by the above described procedure and then successfully scaled up to a pure culture. The photograph of optical microscope and SEM of the strain R-16 were shown in Figure 2a and c, respectively. Microscopic and SEM analysis allowed preliminary identification of the microalga as genus *Scenedesmus*. The strain R-16 was small green colonial microalgae, and the cells were aligned in a flat plate. Most of colonies were composed of 2–4 cells in oblong or ovate shapes, and sometimes unicellular cell can also exist in cultivation. The algal cells approximately ranged from 4 to 16 μm in length and 3 to 8 μm in width depending on different growth stage. Moreover, the selected microalgae with Nile red staining exhibited bright yellow fluorescence, which indicated high intracellular lipid content of the microalgae (Figure 2b).

To further determine the taxonomic position, molecular phylogenetic analysis was used to confirm the isolated strain. The 18S rRNA gene complete sequence of strain R-16 consisting of 1,419 bases was determined and submitted to the GenBank (Accession No.: KC859922). In the phylogram (Additional file 1: Figure S1), the 18S rDNA sequence of strain R-16 confirmed its identification as *Scenedesmus* sp.. The 18S rRNA gene complete sequence of strain R-16 exhibited 100% similarity to that of *Scenedesmus abundans* strain UTEX 343 (Accession No.: X73995.1), and the nucleotides of strain UTEX 343 (1,794 bases) covered all nucleotides of strain R-16 (1,419 bases). However, no identification could be made based on the ITS sequence comparison because the lack of molecular data for the *Scenedesmus abundans* in Genbank. The ITS sequence of strain R-16 shared 99% sequence similarity with that of *Desmodesmus* sp. Tow10/11 T-2 W (Accession No.: DQ417553.1), an uncharacterized *Desmodesmus* specie (Additional file 2: Table S1). Thus, more specific phylogenetic characterization cannot be given, and the microalga was called *Scenedesmus* sp. strain R-16 and used in the following experiments.

### Effects of carbon and nitrogen sources

**Figure 3:**
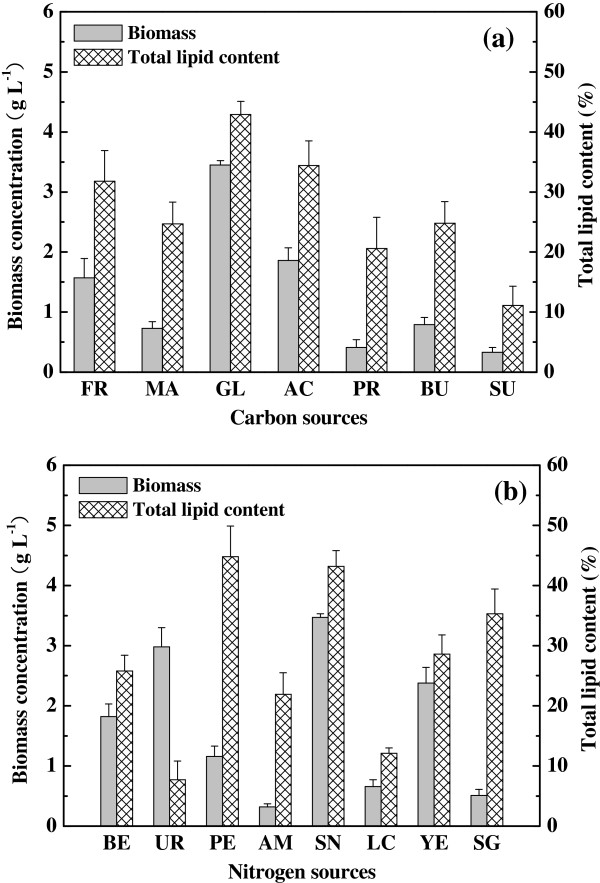
**Biomass concentration and total lipid content of strain R-16 with various carbon and nitrogen sources. (a)**, carbon sources: FR, fructose; MA, maltose; GL, glucose; AC, acetate; PR, propionate; BU, butyrate; SU, sucrose. **(b)**, nitrogen sources: BE, beef extract; UR, urea; PE, peptone; AM, ammonium; SN, sodium nitrate; LC, L-cysteine; YE, yeast extract; SG, sodium glutamate.

Three monosaccharides (fructose, maltose, glucose), three organic acids (acetate, propionate, butyrate) and one disaccharide (sucrose) were used to investigate the influence of carbon source on heterotrophic growth and lipid production in dark condition (Figure 3a and Additional file 3: Figure S2a). Results indicated that the strain R-16 can utilize all the monosaccharides and organic acids but their biomass concentrations and total lipid contents showed great differences. Among above carbon sources, glucose was the best substrate with the maximum biomass concentration (3.46 g L^-1^), specific growth rate (0.819 d^-1^) and highest total lipid content (43.4%). Acetate and fructose were also acceptable carbon sources which exhibited lower total lipid content (34.4%/31.8%), biomass concentration (1.86 g L^-1^/1.57 g L^-1^) and specific growth rate (0.667 d^-1^/0.626 d^-1^), respectively. Further, butyrate and maltose resulted in poor total lipid content (24.8%/24.7%), biomass concentration (0.79 g L^-1^/0.73 g L^-1^) and specific growth rate (0.458 d^-1^/0.438 d^-1^), respectively. Sucrose was not suitable carbon source for strain R-16 since low biomass and total lipid content were acquired. These demonstrated that there was a close relationship between microalgal growth and lipid biosynthesis for heterotrophic strain R-16. Similar law was also observed by some other microalgae species like *Chromochloris zofingiensis*[18,19].

In addition, strain R-16 can utilize both inorganic and organic nitrogen sources for growth and lipid accumulation (Figure 3b and Additional file 3: Figure S2b). Sodium nitrate led to the highest biomass concentration of 3.47 g L^-1^ and specific growth rate of 0.821 d^-1^, followed by that achieved with the medium supplemented with urea (2.98 g L^-1^/0.781 d^-1^) and yeast extract (2.38 g L^-1^/0.727 d^-1^). The maximum total lipid content of 44.8% occurred when peptone was utilized as the nitrogen source, but the biomass concentration and specific growth rate were only 1.16 g L^-1^ and 0.552 d^-1^, respectively. It might be due to that inefficient utilization of peptone could result in the N-starvation of algal cells which induced higher total lipid content of algae [20]. Above results showed that glucose and sodium nitrate were the best carbon and nitrogen source for both growth and lipid production of strain R-16 under the investigated conditions.

### Effect of glucose

**Figure 4:**
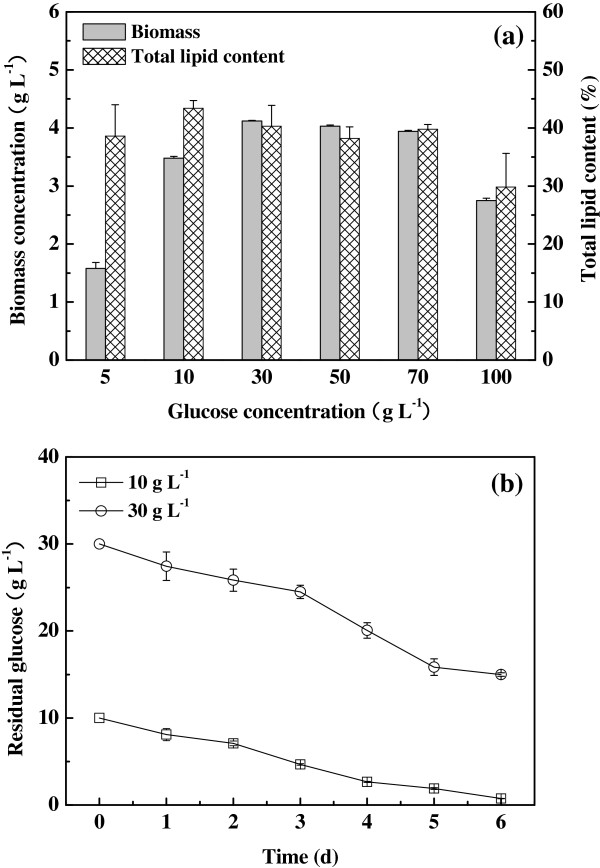
**Effect of glucose. (a)**, Effect of glucose concentration on biomass and total lipid content; **(b)**, Time-course profiles of glucose consumption under various initial glucose concentrations.

Strain R-16 was cultured in the medium containing glucose with various concentrations (5, 10, 30, 50, 70 and 100 g L^-1^). The effects of glucose concentration on algal growth and total lipid content were studied (Figure 4a). When the glucose concentration increased from 5 to 30 g L^-1^, the biomass sharply increased from 1.58 to 4.12 g L^-1^, while further increase of glucose to 100 g L^-1^ led to the decrease of cell concentration. This indicated that excessively high or low glucose in the medium had inhibitory effect on the cell growth. It should be noted that the tolerance boundary for glucose of strain R-16 was higher than those of other algal strains [20]. On the other hand, the total lipid content of 43.4% was obtained with the initial glucose concentration of 10 g L^-1^, and this value was higher than that of 30 g L^-1^ glucose. To determine the optimal glucose concentration, the residual glucose in the culture medium was ascertained as a function of time (Figure 4b). In the culture (6 days) with 10 g L^-1^ glucose, glucose was almost completely utilized and residual glucose was only 0.74 g L^-1^ at the end of the fermentation. This indicated that glucose utilization efficiency reached 92.6%. However, glucose utilization efficiency was just about 50% when 30 g L^-1^ glucose was used as the sole carbon source. The conversion ratio of glucose to oil (*Y*_*oil/glu*_) was an important parameter in the algal biodiesel production [21]. The *Y*_*oil/glu*_ of the culture supplemented with 10 g L^-1^ glucose was 0.162 g g^-1^ of glucose at the end of incubation, which was much higher than that of 30 g L^-1^ glucose (0.111 g g^-1^ of glucose). Considering the above results, although higher biomass concentration appeared at 30 g L^-1^ glucose, the best value of *Y*_*oil/glu*_ was obtained at 10 g L^-1^ glucose. This means that strain R-16 can convert glucose into lipid with high efficiency. Therefore, 10 g L^-1^ glucose was the optimal concentration and was employed in the following tests.

### Effects of sodium nitrate and initial pH

**Figure 5:**
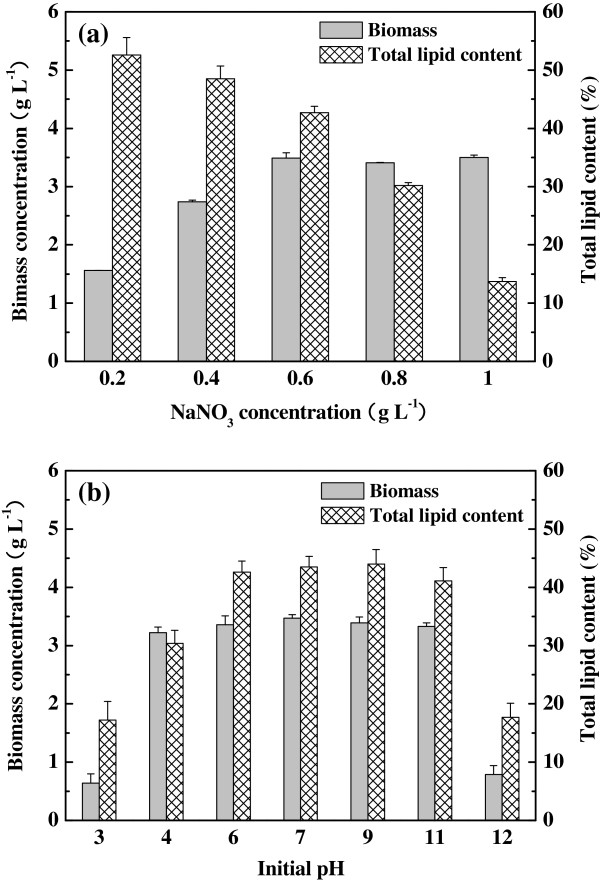
**Effects of NaNO**_**3 **_**and initial pH. (a)**, Effect of NaNO_3_ concentration on biomass and total lipid content. **(b)**, Effect of initial pH on biomass and total lipid content.

Five different NaNO_3_ concentrations (0.2, 0.4, 0.6, 0.8 and 1.0 g L^-1^) were designed to investigate the effect of NaNO_3_ concentration on cell growth and lipid accumulation of stain R-16 (Figure 5a). The algal biomass enhanced significantly when NaNO_3_ concentration increased from 0.2 to 0.6 g L^-1^, while further increase of NaNO_3_ (0.6-1.0 g L^-1^) had little influence on algal growth. This suggested that 0.6 g L^-1^ NaNO_3_ could satisfy the growth requirement of strain R-16. Most importantly, the total lipid content and concentration exhibited significant difference under varied NaNO_3_ concentration. The total lipid content evidently decreased from 52.6% to 13.7% when the NaNO_3_ concentration increased from 0.2 to 1.0 g L^-1^. The result was reasonable because shortage of nitrogen could inhibit the synthesis of protein which was good for the accumulation of lipid in algal cells, whereas the algae did not produce large lipid at high concentration of NaNO_3_[22,23]. Nevertheless, the total biomass and lipid productivity at 0.2 g L^-1^ NaNO_3_ were much lower than those of 0.6 g L^-1^ NaNO_3_, and thus the optimum NaNO_3_ concentration should be 0.6 g L^-1^ by simultaneously considering both the biomass and total lipid content.

The pH was another important factor which had great effect on the properties of microbial surface and flocculation, and such influence can change biochemical metabolism and the form of enzyme system [24]. In the present research the initial pH varied from 3.0 to 12.0 (Figure 5b). This experiment found an interesting phenomenon, that is, the strain R-16 had strong pH tolerance and can grow well in a wide range of pH (4.0-11.0). Furthermore, the total lipid content of algal cells was similar from pH 6.0 to pH 11.0, while the total lipid content decreased at pH 4.0. At two extreme pH (3.0 and 12.0), the algal cells showed poor growth and lipid productivity. These results indicated that the algal growth and lipid accumulation were slightly affected by the pH of the medium (pH 6.0-11.0), which showed the potential of using strain R-16 for the treatment of wastewater or waste biomass into lipid in a wide pH range. In addition, similar phenomenon was also observed by a few researchers with various algal strains (*Chlorella sorokiniana* and *Asterarcys quadricellulare*) in autotrophic situation [20,25]. However, in this study the strain R-16 can grow well in heterotrophic culture condition and also exhibited amazing pH tolerance, which might be caused by self-metabolism regulation or special structure of the microalgae. Nevertheless, up to now, the mechanism of this process has not yet been given, and the detailed explanation needed to be further investigated in the future work.

### Growth and lipid production under various culture modes

**Figure 6:**
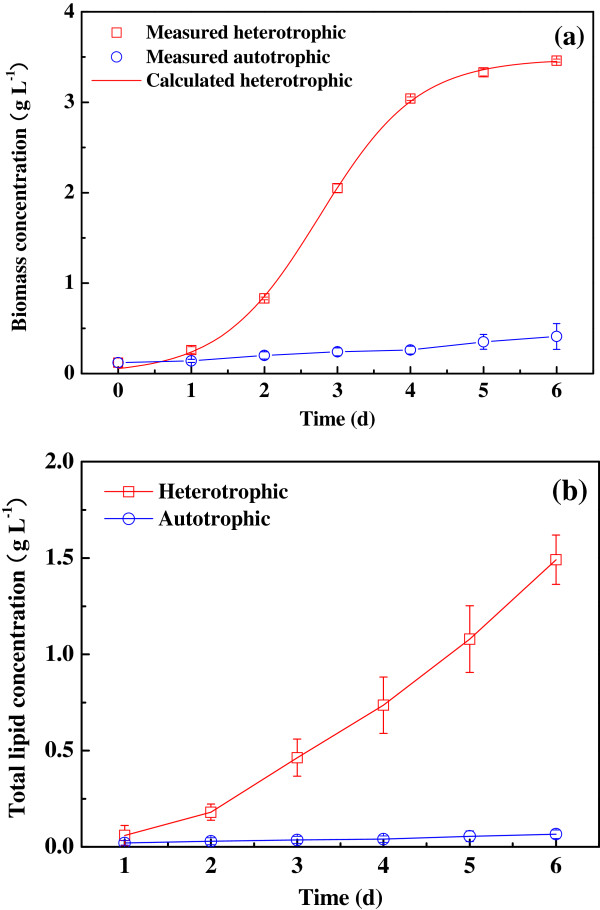
**Growth curves and profiles of lipid accumulation. (a)**, Growth curves of strain R-16 under autotrophic and heterotrophic conditions; **(b)**, Lipid accumulation of strain R-16 under autotrophic and heterotrophic conditions.

Besides heterotrophic cultivation, strain R-16 can grow in autotrophic conditions as well (Figure 6). The biomass of autotrophic microalgae reached 0.41 g L^-1^ in 6 days growth, whereas the biomass of heterotrophic microalgae increased to 3.46 g L^-1^ in the same experimental period. By fitting the measured data of biomass concentration to time (Figure 6a), the *X*_*max*_ of heterotrophic culture obtained from Logistic model was 3.48 g L^-1^, which was similar to the experimental data of 3.46 g L^-1^. This value was much higher than that of autotrophic algae (0.41 g L^-1^). In addition, correlation coefficient (*R*^*2*^) for the model fit was 0.999, indicating distinguished agreement of Logistic model to experimental results. The results showed that heterotrophic culture of strain R-16 exhibited higher growth rate and biomass concentration in comparison with its autotrophic culture. On the other side, the cumulative rate of lipid of strain R-16 under heterotrophic culture was faster than that under autotrophic culture (Figure 6b). The maximum total lipid concentration of strain R-16 was 1.50 g L^-1^ in heterotrophic culture, but the total lipid concentration in autotrophic culture was only 0.07 g L^-1^ on the sixth day. Heterotrophic algal cells were rich in lipid compounds, whereas autotrophic strain R-16 accumulated little lipid in the whole cultivation period. In a study, similar result was also found in the cultivation of *Chlorella* cells [12]. Moreover, the heterotrophic algae grew in lag phase (about 1 day) to adapt themselves to the environmental conditions, and entered the exponential growth phase and grew exponentially until the fourth day. And then, algal growth became slow and turned into stationary phase (5–6 days). It can be found the variation of lipid concentration correlated with the cell growth in the exponential phase, which indicated that the increase of biomass concentration resulted in the increase of total lipid concentration. By contrast, during stationary phase the algal growth was slow and most of lipid came from the accumulation of microalgae. Above results showed that heterotrophic culture of strain R-16 was better than autotrophic culture in terms of cell growth and lipid production.

### Comparison of lipid production capability with relevant studies

The biomass and lipid production ability of different microalgal strains using glucose as carbon source were summarized (Table 1). It was shown that the biomass of 3.46 g L^-1^, total lipid content of 43.4% and lipid productivity of 250.27 mg L^-1^ d^-1^ attained in the present work were comparable or even higher than those reported in literature. Furthermore, it was worth noting that the *Y*_*oil/glu*_ of this research (0.162 g g^-1^) was higher than the previous reports of *Chlorella sorokiniana* (0.057 g g^-1^) and *Scenedesmus quadricauda* (0.15 g g^-1^) (Table 1). This indicated that the strain R-16 can efficiently convert the substrate to cellular lipid. Moreover, it was proposed that the growth rate and lipid productivity in mixotrophic culture conditions were higher in comparison with those in autotrophic and heterotrophic conditions [13]. Compared to heterotrophic culture, *Chlorella protothecoides* in mixotrophic culture can produce 69% higher lipid with 61.5% less release of CO_2_[21]. Additionally, Cheirsilp and Torpee [13] found that freshwater *Chlorella* sp., marine *Chlorella* sp., *Nannochloropsis* sp. and *Cheatoceros* sp. can accumulate high lipid content in photoautotrophic, heterotrophic and mixotrophic cultures. The highest biomass and lipid production were all obtained in mixotrophic culture for the four algal strains. Moreover, the lipid content of *Scenedesmus quadricauda* was 33.1% in mixotrophic culture, whereas the lipid content decreased to 14-28% in photoautotrophic and heterotrophic cultures [28]. In this work, the total lipid content of strain R-16 reached 43.4% in heterotrophic culture, indicating that the biomass and lipid production capacity of strain R-16 could be further improved by using the mixotrophic culture.

**Table 1:** Comparison of biomass and lipid production with glucose as carbon source

**Microalgae**	**Glucose concentration (g L**^**-1**^**)**	**Biomass concentration (g L**^**-1**^**)**	**Total lipid content (%DW)**	**Lipid productivity (mg L**^**-1 **^**d**^**-1**^**)**	***Y***_***oil/glu***_	**Reference**
*Chlorella sorokiniana* Shih.et Krauss MIC-G5	1	0.72^a^	33	29.8	NA	[26]
*Chlorella* sp.	10	3.76	15.6	58.8	NA	[13]
*Chlorella vulgaris* C4-3	10	0.8	15.78	21^a^	NA	[27]
*Chlorella saccharophila* UTEX 247	40	1.1	37	58.5^a^	NA	[12]
*Chlorella sorokiniana* CCTCCM209220	40	3.2	56	256	0.057^a^	[20]
*Ettlia texensis*	3	1.2^a^	14-19	NA	NA	[11]
*Nannochloropsis* sp.	10	3.83	19.3	74	NA	[13]
*Monoraphidium* sp. FXY-10	10	3.96	37.56	148.74	NA	[8]
*Scenedesmus quadricauda*	5	3.39	22.1^a^	107.1^a^	0.15^a^	[28]
R-16	10	3.46	43.4	250.27	0.162	This study

^a^Calculated value according to reported data.

In addition, a critical challenge of heterotrophic microalgae derived from the high cost of culture medium, especially the carbon sources [28]. Most researchers reduced the cost of algal cultivation by using some cheap and easily available feedstock (such as starch, wastewater and cellulose-hydrolyzed solution) and gained promising results [29,30]. It was found that some microalgae like strain R-16 can utilize the volatile fatty acids (VFAs) as the substrate for growth and lipid accumulation [8]. In the production of other bio-energy (such as bio-hydrogen, bio-methane, bio-ethanol, bio-butanol and bio-electricity), there are many VFAs left in the effluent [31-33]. Take bio-hydrogen production for example, the degradation of large molecular substrates by dark-fermentative bacteria can generate many VFAs, which mainly contain acetic acid, propionic acid and butyric acid [34]. However, VFAs in effluent may decrease the pH of reaction system and induce inhibition effect to the microorganisms or pollution to the environment. Photo-fermentative bacteria can utilize the VFAs from dark-fermentation as electron donors and produce hydrogen in the presence of light [35]. Currently, most studies focused on the combination of dark- and photo-fermentation to alleviate the end-product inhibition and improve the substrate conversion efficiency [34-36]. Though the combined system can markedly increase the theoretical hydrogen yield to 12 mol mol^-1^ glucose^-1^, the experimental energy conversion efficiency of the combined system is still not high enough for the practical reality of biological hydrogen production [37]. Furthermore, it should be noted that the photo-fermentative bacteria need additional light with low photosynthetic efficiency (less than 10%), which could greatly increase the operating cost [38]. By contrast, above mentioned VFAs in effluent can be further utilized by microalgae without light for lipid production and energy recovery, whereas so far related research on this field was lacking. So, it was a promising strategy to combine the microalgal lipid production with one or more other bio-energy production methods to reduce the cost of algal cultivation and increase the energy recovery of the whole process.

## Conclusions

The newly developed ultrasonic-assisted Nile red method was applied in the screening of lipid-rich microalgae and proved to be an efficient isolation procedure. Based on this method, a novel green microalgal strain, *Scenedesmus* sp. R-16, was isolated and characterized from 88 microalgae isolates. Strain R-16 can be acclimatized to high glucose (up to 100 g L^-1^) and a wide range of pH (4.0-11.0). Many carbon and nitrogen sources can be used by strain R-16 for growth and lipid production. By using the optimum carbon source (10 g L^-1^ of glucose) and nitrogen source (0.6 g L^-1^ of sodium nitrate), strain R-16 exhibited high biomass of 3.46 g L^-1^and total lipid content of 43.4% under heterotrophic condition. When nitrogen was scarce, total lipid content could reach a maximum value of 52.6%. Compared with other reported oleaginous algal strains, *Scenedesmus* sp. R-16 showed great potential in the lipid production for renewable biodiesel production.

## Methods

### Isolation and purification of the microalga

Samples of soil were collected from Harbin, Heilongjiang province, China, and then were inoculated in autoclaved BG11 medium at 25 ± 1°C under cool white fluorescent light until algal growth was detected [39]. The pre-cultured samples were diluted and streaked on BG11 medium-enriched agar plates. Individual colonies were picked up and cultured in liquid BG11 medium that contained glucose using the same culture conditions described above. The streaking and inoculation procedures were repeated about 3–4 times until pure cultures were attained.

### Molecular identification

Genomic DNA was extracted with a SK1375 kit according to the manufacturer’s instructions [40]. The 18S rDNA and ITS regions were amplified using the primers described in Additional file 4: Table S2 [41,42]. The 25 μl PCR reaction system contained approximately 1 μl of template DNA, 0.5 μl (10 μmol L^-1^) of forward primer, 0.5 μl (10 μmol L^-1^) of reverse primer, 0.5 μl dNTP mixer (10 mmol L^-1^ each), 1.0 U of Ex Taq DNA polymerase and 2.5 μl 10 × Ex Taq PCR buffer. Amplification conditions were performed as follows: 5 min at 94°C, followed by 35 cycles of denaturation at 94°C for 30 s, 35 s annealing at 55°C, and 1 min extension step at 72°C with a final extension of 8 min at 72°C. The PCR products were separated by electrophoresis on 1.0% agarose gel. Afterwards, bands were extracted from gel and purified using a PCR purification kit (SK1131 kit). The purified PCR products were ligated into the pUCm-T followed by transforming into *Escherichia coli* competent cells (SK2301 kit). Recombinant plasmid was extracted from transformed *E. coli* with a SK1191 kit following the guidelines of the manufacturer [43]. All kits were provided by Sangon Biotech (Shanghai) Co., Ltd., (Shanghai, China). Sequence alignment and analysis of the similarity of the18S rRNA and ITS gene were performed with BLAST in GenBank database.

### Culture conditions

In the autotrophic culture, algal cells in the stationary phase were inoculated into 250 ml Erlenmeyer flasks containing 150 ml BG11 medium, which had been adjusted to near neutral pH and autoclaved at 121°C for 15 min. The microalgae were cultured at 25 ± 1°C using incubator shaker at 130 rpm with serial florescence light of around 5000 lux. For heterotrophic culture, seven carbon sources (fructose, maltose, glucose, acetate, propionate, butyrate, sucrose) were separately supplemented to the liquid culture medium. The initial concentrations of all carbon sources were calculated as the same carbon atom number of 10 g L^-1^ glucose. Beef extract, urea, peptone, ammonium chloride, sodium nitrate, L-cysteine, yeast extract and sodium glutamate were chosen as the nitrogen sources with the initial concentrations computed as the same nitrogen atom number of 0.6 g L^-1^ sodium nitrate. The glucose concentration, sodium nitrate concentration and initial pH were in the range of 5–100 g L^-1^, 0.2-1.0 g L^-1^ and 3.0-12.0, respectively. Each culture was cultured using the same conditions described in the autotrophic culture without light exposure. The initial algal concentration of both autotrophic and heterotrophic trials was approximately 0.1 g L^-1^. After reaching the stationary phase (defined by no further increase in cell concentration), the cultures were harvested and analyzed for biomass and lipid production. All experiments were conducted in triplicate, and results were expressed as means of the replicates along with standard deviation (± SD).

### Nile red staining

Eighty-eight algal strains that had high biomass were chosen for the determination of lipid content. Based on preliminary procedure for improved Nile red staining (data not published), the selected cells (5 ml) were centrifuged at 8,000 g for 10 min and washed with distilled water several times. Then the collected cells were re-suspended in a 10 ml tubes and pretreated with an ultrasonic processor (Sonics VCX130PB, USA). Furthermore, 15 μl of Nile red solution (0.5 mg ml^-1^ in acetone) was added to 5 ml of algal suspensions and gently vortexed for 1 min. After 15 min of incubation in darkness, the fluorescence of the suspension was measured with a fluorescence spectrophotometer (JASCO FP-6500, Japan). According to preliminary experiments, the excitation and emission wavelengths for the fluorescence determination were selected as 530 and 568 nm, respectively. Unstained cells and Nile red alone were used as the autofluorescence control. The relative fluorescence intensity of Nile red was attained after subtraction of both the autofluorescence of microalgae and the self-fluorescence of Nile red.

### Algal growth kinetics

Most growth processes of microorganisms can be described by Monod, Baranyi and Logistic equations [30,44]. Among them, Logistic equation was widely applied to interpret the relationship between the algal growth and biomass density in nutrition-limited conditions [45]. Thus, Logistic model Eq. (1) was chosen for algal growth.

(1) $$\frac{\mathrm{dX}}{\mathrm{dt}}=\mathrm{kc}\left(1-\frac{X}{X_{\max}}\right)X$$

where *dX*/*dt* was the microalgal growth rate; *k*_*c*_ was the maximum specific growth rate of the microalgae, *X* was the biomass concentration of microalgae, *X*_*max*_ was the maximum cell concentration.

At the beginning of the experiments (*t* = 0), the initial algal concentration was *X = X*_*0*_ and after integration the expression of Logistic equation became Eq. (2):

(2) $$X=\frac{X_{0}X_{\max}e^{\mathrm{Kct}}}{X_{\max}-X_{0}+X_{0}e^{\mathrm{Kct}}}$$

### Analytical methods

The cell morphology of the isolated strain was observed using microscope and scanning electron microscopy (SEM). Light and fluorescence micrographs were obtained with a microfluorometer (BX51-TF, Olympus Optical Co., Ltd., Japan). Before analyzing with SEM, the surface of algal cells was coated with a gold layer by employing Sputter Coater (Hitachi E-1010, Japan). Further, the treated specimens were explored by a scanning electron microscope (Hitachi S-3400 N, Japan) as the methods described previously [46]. The biomass in dry weight (DW) and total lipid content were determined as the produces described previously [20,47,48]. The residual glucose in the culture broth was measured with oxidase method [35]. The pH value was monitored by a pHS-3C pH meter (Shanghai Leici Instrument Factory, China). The light intensity was measured on the surface of flasks with a digital luxmeter (TES-1332A, TES Electrical Electronic Co., China). The specific growth rate (*μ*, d^-1^) of microalgae was calculated according to the Eq. (3):

(3) $$\mu =\frac{\mathrm{In}\;N_{2}-\mathrm{In}\;N_{1}}{t_{2}-t_{1}}$$

where *N*_*2*_ and *N*_*1*_ represented the DW values at the time *t*_*2*_ and *t*_*1*_, respectively.

## Abbreviations

SEM: Scanning electron microscopy; 18S rRNA: 18S ribosomal ribonucleic acid; 18S rDNA: 18S ribosomal deoxyribonucleic acid; ITS: Internal transcribed spacer; N: Nitrogen; Yoil/glu: Conversion ratio of glucose to oil; NaNO3: Sodium nitrate; CO2: Carbon dioxide; VFAs: Volatile fatty acids; DNA: Deoxyribonucleic acid; PCR: Polymerase chain reaction; BLAST: Basic local alignment search tool; SD: Standard deviation; DW: Dry weight; NA: Not available.

## Competing interests

The authors declare that they have no competing interests.

## Authors’ contributions

HYR designed the study, executed the experimental work, data interpretation and drafted the manuscript. BFL participated in experimental design and data interpretation, and reviewed the manuscript. CM assisted the laboratory work. LZ commented on the manuscript. NQR contributed to the design of the study, data interpretation and reviewed the manuscript. All authors have read and approved the final manuscript.

## Supplementary Material

Additional file 1: Figure S1 Phylogenetic analysis of strain R-16 and its closely related species based on 18S rRNA gene sequences in Genbank. Click here for file

Additional file 2: Table S1 Results from BLAST searches using the 18S rDNA and ITS sequences of strain R-16. Click here for file

Additional file 3: Figure S2 Specific growth rates of strain R-16 with various (a), carbon sources and (b), nitrogen sources. Click here for file

Additional file 4: Table S2 Primers for genomic DNA amplification. Click here for file
