# Supplementary material for: A new lipid-rich microalga Scenedesmus sp. strain R-16 isolated using Nile red staining: effects of carbon and nitrogen sources and initial pH on the biomass and lipid production
Source: Biotechnol Biofuels. 2013 Oct 6;6:143. doi: 10.1186/1754-6834-6-143 (PMC3853715; doi:10.1186/1754-6834-6-143)
Supplement: Additional file 1: Figure S1 — Phylogenetic analysis of strain R-16 and its closely related species based on 18S rRNA gene sequences in Genbank. [file 1754-6834-6-143-S1.docx]

*Scenedesmus* sp. Lake Las Vegas (JX910112.1)

*Scenedesmus armatus* CCAP 276/4A (FR865727.1)

*Scenedesmus subspicatus* UTEX 2532 (AJ249514.1)

*Scenedesmus abundans* UTEX 343 (X73995.1)

R-16 (KC859922)

*Desmodesmus communis* AICB 141 (JQ922412.1)

*Scenedesmus communis* UTEX 76 (X73994.1)

*Scenedesmus costato* SAG 18.81 (X91265.1)

*Scenedesmaceae* sp. Tow 9/21 P-13w (AY197638.1)

*Chlorella emersonii* CCAP 211/11M (FR865657.1)

100

99

79

99

75

61

64

0.005

Figure S1
