# Supplementary material for: A new lipid-rich microalga Scenedesmus sp. strain R-16 isolated using Nile red staining: effects of carbon and nitrogen sources and initial pH on the biomass and lipid production
Source: Biotechnol Biofuels. 2013 Oct 6;6:143. doi: 10.1186/1754-6834-6-143 (PMC3853715; doi:10.1186/1754-6834-6-143)
Supplement: Additional file 2: Table S1 — Results from BLAST searches using the 18S rDNA and ITS sequences of strain R-16. [file 1754-6834-6-143-S2.docx]

| Marker gene | Closest match | Genbank accession number and  sequence length (bp) | Sequence length of  R-16 (bp) | Query coverage  (%) | Similarity  (%) |
| --- | --- | --- | --- | --- | --- |
| 18S rDNA | *Scenedesmus abundans*  UTEX 343 | X73995.1, 1,794 | 1,419 | 100 | 100 |
| ITS | *Desmodesmus* sp. Tow10/11T-2W | DQ417553.1, 627 | 636 | 97 | 99 |

**Table S1** Results from BLAST searches using the 18S rDNA and ITS sequences of strain R-16
