# Supplementary material for: A new lipid-rich microalga Scenedesmus sp. strain R-16 isolated using Nile red staining: effects of carbon and nitrogen sources and initial pH on the biomass and lipid production
Source: Biotechnol Biofuels. 2013 Oct 6;6:143. doi: 10.1186/1754-6834-6-143 (PMC3853715; doi:10.1186/1754-6834-6-143)
Supplement: Additional file 4: Table S2 — Primers for genomic DNA amplification. [file 1754-6834-6-143-S4.docx]

**Table S2** Primers for genomic DNA amplification

| Primer | Sequence 5’ → 3’ |
| --- | --- |
| NS1 | GTAGTCATATGCTTGTCTC |
| NS6 | GCATCACAGACCTGTTATTGCCTC |
| ITS1 | TCCGTAGGTGAACCTGCGG |
| ITS4 | TCCTCCGCTTATTGATATGC |
